# A first-in-human pilot study of Wharton’s jelly-derived mesenchymal stem cell injection for urethral stricture: safety and preliminary efficacy

**DOI:** 10.1007/s00345-026-06414-0

**Published:** 2026-04-14

**Authors:** Mazhar Ortac, M. Firat Ozervarli, Seyfettin Anil Tantekin, Rifat Burak Ergul, Yasin Ates, Arda Tunc Aydinoglu, Resat Aydin, Senol Tonyali, Anthony Atala

**Affiliations:** 1 Department of Urology, Istanbul Faculty of Medicine, Istanbul University, Istanbul, Turkey; 2 Department of Urology, Haseki Training and Research Hospital, Istanbul, Turkey; 3 Department of Urology, Wake Forest University, Winston Salem, NC USA

**Keywords:** Urethral stricture, Mesenchymal stem cells, Wharton’s jelly, Regenerative therapy, Pilot study

## Abstract

**Background:**

Urethral stricture is a fibrotic narrowing of the urethra with limited long-term success using minimally invasive treatments. Mesenchymal stem cells (MSCs) possess antifibrotic and regenerative properties that may offer a novel therapeutic approach. This first-in-human pilot study evaluated the safety and preliminary efficacy of Wharton’s jelly-derived MSC injections for recurrent bulbar urethral stricture.

**Methods:**

Eleven men with recurrent bulbar urethral strictures ≤ 2 cm underwent urethral dilatation followed 3–5 days later by intralesional injection of 4 × 10^6 MSCs. MSCs were cryopreserved under ultra-low temperatures, transported at 2–8 °C on the day of injection, and viability (> 70%) was confirmed post-thaw. Patients were followed for six months, with assessments including uroflowmetry, post-void residual, urethroscopy or retrograde urethrography, and patient-reported outcomes (IPSS, IIEF, USS-PROM). Safety was monitored throughout.

**Results:**

The procedure was completed in all patients without technical difficulties. No systemic or long-term local adverse events were observed. Two patients experienced transient dysuria and one had minimal hematuria, all resolving spontaneously. Median Qmax improved from 8.9 mL/s preoperatively to 16.4 mL/s at 1 month but declined toward baseline by 6 months. Patient-reported outcomes showed short-term improvements, with significant reductions in IPSS and USS-PROM scores at 1 and 3 months. No statistically significant changes were observed in uroflowmetry parameters over the six-month follow-up.

**Conclusions:**

Intralesional injection of Wharton’s jelly-derived MSCs following urethral dilatation is safe and feasible in humans. While objective urinary flow improvements were limited, patient-reported symptoms showed short-term benefit. These findings support further investigation in larger, controlled trials to optimize dosing, evaluate repeated administration, and determine efficacy in urethral stricture management.

## Introduction

Urethral stricture is defined as a fibrotic narrowing of any segment of the urethra involving the surrounding corpus spongiosum [1]. Current treatment options vary according to the location and severity of the stricture and include minimally invasive endoscopic approaches, such as urethral dilatation and internal urethrotomy, as well as open reconstructive procedures, most notably urethroplasty [2]. Endoscopic techniques are widely used as first-line interventions due to their minimally invasive nature; however, they are associated with high recurrence rates [3]. In contrast, urethroplasty demonstrates durable success rates exceeding 85% across different techniques [4]. Nevertheless, urethroplasty requires more extended hospitalization, prolonged catheterization, and carries the risk of complications, including ejaculatory dysfunction, reduced glans tumescence, penile shortening, and chordee, depending on the technique applied [5–7].

In recent years, alternative adjunctive treatments such as intralesional steroids, mitomycin C, platelet-rich plasma, optilume drug-coated balloons, halofuginone, and endourethral brachytherapy have been reported; however, none have yet proven to be adequate substitutes for standard therapies [8–11].

Given the limitations of existing treatment modalities, regenerative medicine strategies, including stem cell therapy and tissue engineering, are attracting increasing attention as potential future options [12]. Recent reviews have emphasized the translational challenges of tissue engineering approaches for urethral reconstruction, which are not yet ready for routine clinical application [13]. Mesenchymal stem cells (MSCs) exhibit potent antifibrotic activity through various mechanisms, including immunomodulation, inhibition of the TGF-β1 signaling pathway, mitigation of hypoxia, regulation of oxidative stress, and remodeling of the extracellular matrix [14]. Given that fibrosis plays a central role in the pathophysiology of urethral stricture, it was hypothesized that the antifibrotic properties of MSCs could be harnessed to reduce scar formation and thereby contribute to the treatment of this disease [15].

MSCs are multipotent adult stem cells present in bone marrow, adipose tissue, umbilical cord, and amniotic membrane. Through their trophic and immunomodulatory functions, they play a central role in promoting tissue regeneration [16].

Consequently, MSC-based therapies are under investigation in various fibrotic and ischemic diseases, including myocardial infarction, idiopathic pulmonary fibrosis, liver cirrhosis, renal fibrosis, Peyronie’s disease, and erectile dysfunction [17]. Based on these regenerative and antifibrotic properties, MSC therapy is hypothesized to have therapeutic potential in urethral stricture disease, which is fundamentally a fibrotic disorder. Intralesional MSC injection following urethral dilatation may prevent stricture recurrence or prolong the recurrence-free interval, thereby reducing the need for repeated dilatations and minimizing healthcare utilization. Although preclinical studies have shown encouraging results in experimental models of urethral stricture, no clinical studies have yet evaluated the use of stem cell therapy in this context. Therefore, the present study was designed as a first-in-human pilot trial to assess the safety and preliminary efficacy of MSC injections for the management of urethral stricture.

## Materials and methods

### Study design and patients

This study was designed as a prospective, single-arm, self-controlled interventional trial conducted between May 2024 and August 2025 at a single center. Eligible patients were men aged 18 years or older with a bulbar urethral stricture measuring ≤ 2 cm. All included patients had undergone at least one prior dilatation and experienced recurrence.

Exclusion criteria were multiple strictures, obliterative strictures, strictures that developed after prostatectomy, and a history of open urethral surgery.

Before treatment, the diagnosis of urethral stricture was confirmed using uroflowmetry (UF), urethroscopy, and urethrography. Patients also underwent urinalysis (UA) and post-void residual (PVR) testing. To assess patient satisfaction and symptom burden, validated questionnaires were administered, including the International Prostate Symptom Score (IPSS), the International Index of Erectile Function (IIEF), and the Urethral Stricture Surgery-Patient-Reported Outcome Measure (USS-PROM, validated version) [18]. Demographic characteristics, structural etiology, and treatment-related complications were also recorded at each visit.

The study was approved by the Ethics Committee of Istanbul Faculty of Medicine (Approval No: 2011-KAEK-57-1775). All procedures were conducted in accordance with the principles of the Declaration of Helsinki, applicable national regulations, and the Good Clinical Practice guidelines (ISO 14155:2011). The trial was also prospectively registered with the Turkish Ministry of Health (Registration No: 234256661). Written informed consent was obtained from each participant before enrollment.

### Mesenchymal stem cell isolation

Umbilical cord tissue was aseptically collected from a consenting donor after negative serological screening for HIV-1/2, HBV, HCV, *Treponema pallidum*, and HTLV-1/2, in accordance with international tissue banking standards [19]. The umbilical cord tissue was transported in a tissue preservation solution and processed under current Good Manufacturing Practice (cGMP) conditions at the STEMBIO Cell and Tissue Technologies Laboratory in Kocaeli, Türkiye. Upon arrival, the cord was thoroughly rinsed with isotonic 0.9% NaCl solution. The vessels were carefully dissected using a scalpel and forceps, and the Wharton’s jelly was cut into small fragments. Mesenchymal stem cells (MSCs) were isolated using the explant culture method.

The isolated MSCs were expanded in a xeno-free, chemically defined medium consisting of MSC NutriStem XF Basal Medium (Sartorius, Göttingen, Germany), NutriStem XF Supplement (Sartorius, Göttingen, Germany), 2% GMP-grade human serum, and antibiotics applied only during initial processing [20]. Cell number and viability were assessed after each passage using light microscopy (ZEISS Primostar 3, Zeiss, Germany) and an automated cell counter (ADAM MC, NanoEntek, USA). Cultures were then expanded up to passage 3.

Characterization was performed on MSCs at passage 3. Cell number and viability were confirmed using automated cell counting, and immunophenotyping was analyzed by flow cytometry (Navios, Beckman Coulter, USA). The cells were positive for CD90, CD105, CD44, and CD73, and negative for CD34, CD45, CD11b, CD14, CD19, CD79a, and HLA-DR. This immunophenotype met the International Society for Cell and Gene Therapy (ISCT) minimal criteria for MSCs (≥ 95% expression of positive markers and ≤ 2% expression of negative markers) [21].

Release specifications included viability ≥ 70%, sterility according to USP < 71> (absence of microbial growth), endotoxin ≤ 5 EU/kg according to USP < 85>, and a negative mycoplasma PCR [22]. Identity was confirmed by flow cytometry. Exploratory potency testing, including IDO-1 and PD-L1 expression after IFN-γ licensing, was also performed.

Following successful quality control, MSCs were cryopreserved in vials as ready-to-use suspended products. Each vial was stored under monitored conditions at ultra-low temperatures (–80 °C or in liquid nitrogen), labeled with the donor lot number, passage number, and total cell dose. On the day of implantation, vials were transported at 2–8 °C, thawed, and post-thaw viability was reassessed immediately before injection to ensure greater than 70% survival [23].

### Surgical technique

Three to five days before stem cell injection, all patients underwent urethral dilatation using S-curve dilators (Cook Medical, Bloomington, IN, USA). Under direct visualization, a guidewire was carefully passed through the urethral stricture. Sequential filiform dilators were then advanced over the guidewire, starting from 6 F and gradually progressing to 18 F, to achieve adequate dilation.

**Fig. 1:**
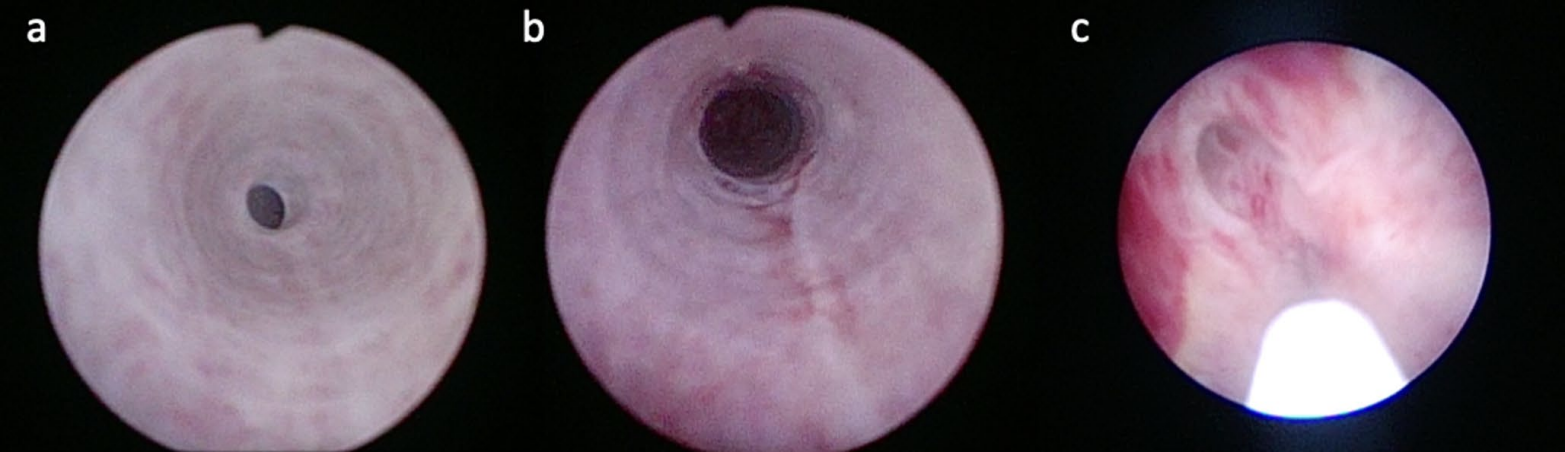
(**a**) Bulbar urethral stricture before treatment. (**b**) Lumen after sequential dilatation. (**c**) Intralesional MSC injection under cystoscopic guidance

Stem cell administration was performed using a rigid 7 Fr pediatric cystoscope. In each patient, a 1 mL isotonic solution containing 1 × 10^6 mesenchymal stem cells were injected into each of the four quadrants of the bulbar urethral stricture, corresponding to the 1, 5, 7, and 11 o’clock positions, via a cystoscopic injection needle (Cook Williams Needle, Cook Medical, Bloomington, IN, USA- Fig. 1).

Following injection, patients were observed for potential complications, including bleeding, urinary obstruction, and allergic reactions, particularly during the first two postoperative hours. All procedures were performed by a single experienced urological surgeon. The majority were conducted under local anesthesia, while a minority were performed under general anesthesia, depending on patient preference and clinical considerations.

This dosing strategy (1 × 10^6 cells per quadrant; total 4 × 10^6 cells) was selected based on prior reports supporting effective local tissue repair with MSC densities in the range of approximately 1–5 × 10^6 cells/cm², while remaining within the established clinical safety margins of MSC therapy [16, 20, 24].

### Follow-up

The effectiveness of treatment was evaluated at follow-up visits scheduled at 1, 3, and 6 months after the procedure. At each visit, assessments included uroflowmetry, urethroscopy or retrograde urethrography, post-void residual testing, and completion of validated questionnaires: the IPSS, the IIEF-5, and the USS-PROM.

Additionally, at every follow-up visit, patients were systematically questioned about potential systemic and local adverse events, and all findings were thoroughly documented.

During each follow-up, the mean values of maximum flow rate (Qmax), mean flow rate (Qmean), voided volume, PVR, IPSS, and USS-PROM were compared with baseline preoperative measurements to evaluate functional and symptomatic outcomes.

### Study objectives and assessment

The primary objectives of this pilot study were to assess the safety and feasibility of intralesional mesenchymal stem cell (MSC) injection following urethral dilatation and to explore preliminary evidence of efficacy. Safety was assessed through the occurrence of procedure-related or systemic adverse events, which were carefully monitored and documented at each follow-up visit. Efficacy was evaluated by changes in uroflowmetry parameters Qmax and mean urinary flow rate, post-void residual volume, urethroscopic or radiographic findings, and validated patient-reported outcomes, including the IPSS, the IIEF, and the USS-PROM.

## Results

The mean age of the 11 patients included in the study was 39.8 years (range, 19–72 years). Two patients had coronary artery disease, and three had hypertension. The duration of the injection procedure ranged from 3 to 6 min.

**Fig. 2:**
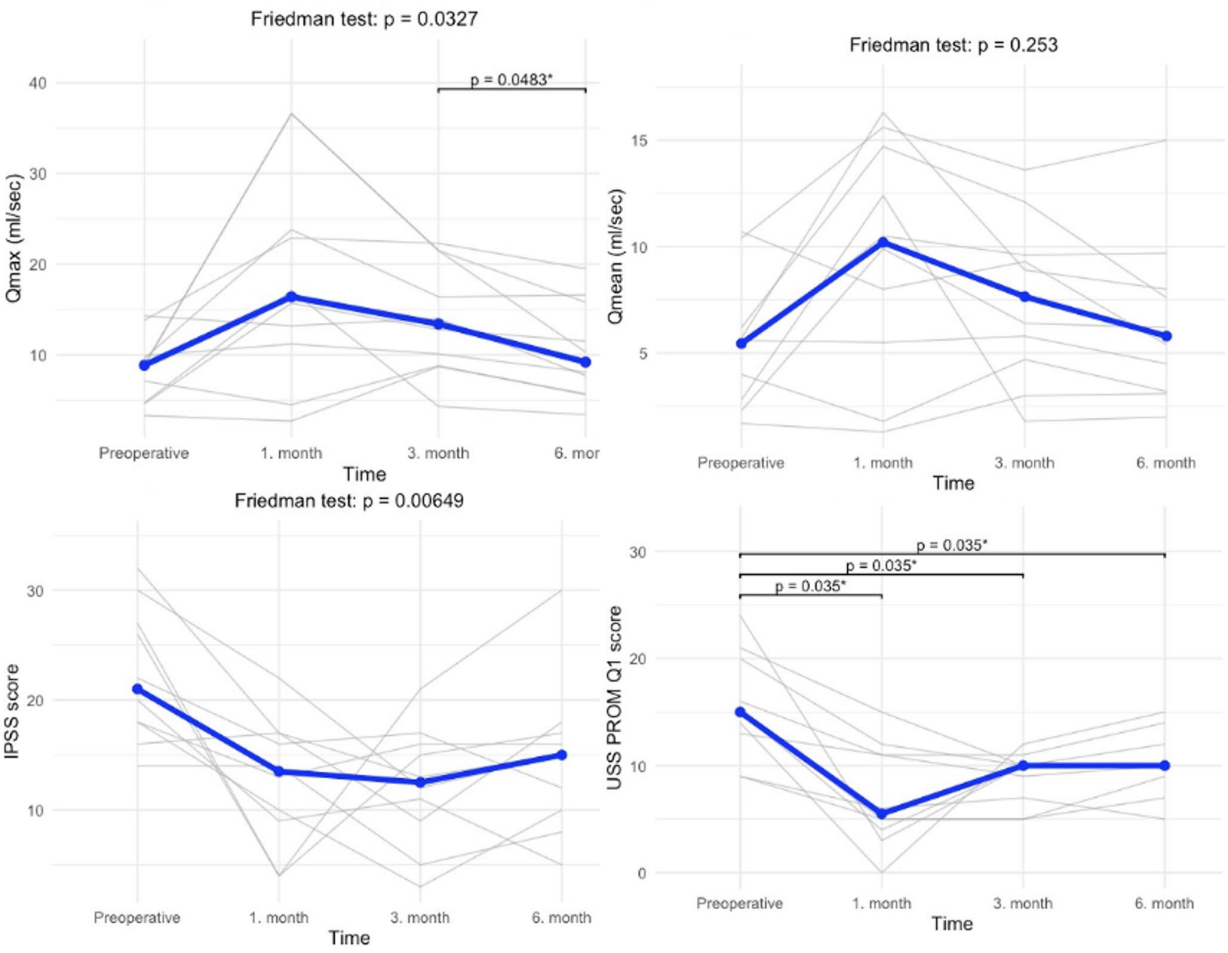
This figure shows the 6-month follow-up changes in Qmax, Qmean, IPSS, and USS PROM scores from the preoperative period to postoperative time points

Median maximum urinary flow rate values were 8.9 mL/s (5.3–9.8) preoperatively, 16.4 mL/s (11.7–23.6) at 1 month, 13.4 mL/s (9.1–20.2) at 3 months, and 9.2 mL/s (6.2–14.7) at 6 months. Mean urinary flow rate values were 5.4 mL/s (3.1–6.1) preoperatively, 10.2 mL/s (6.1–14.1) at 1 month, 7.7 mL/s (5.0–9.5) at 3 months, and 5.8 mL/s (3.5–7.9) at 6 months. No statistically significant improvements were observed in either Qmax or Qmean values when comparing preoperative and postoperative measurements (Fig. 2).

The IPSS was 21.0 (18.0–26.8) preoperatively, 13.5 (9.2–16.8) at 1 month, 12.5 (9.5–15.8) at 3 months, and 15.0 (10.5–16.8) at 6 months. A statistically significant improvement was observed between baseline and the third month (*p* = 0.035), but started to rise again by 6 months.

The total USS-PROM score for lower urinary tract symptoms (0 = least symptomatic, 24 = most symptomatic) was 15.0 (13.2–19.0) preoperatively, 5.5 (4.2–11.0) at 1 month, 10.0 (7.5–10.0) at 3 months, and 10.0 (9.2–11.5) at 6 months. Statistically significant improvements were observed at 1, 3, and 6 months compared with the preoperative period (*p* = 0.035).

The USS-PROM voiding picture score (1 = best, 4 = worst) was 3.0 (3.0–3.0) preoperatively, 2.0 (2.0–2.8) at 1 and 3 months, and 3.0 (2.0–3.0) at 6 months. No statistically significant differences were found in pairwise comparisons (all *p* ≥ 0.088).

The USS-PROM general health status based on the Visual Analog Scale (100 = best possible health, 0 = worst) showed median values of 45% (36.2–72.5) preoperatively, 77.5% (64.0–87.5) at 1 month, 70% (52.5–80.0) at 3 months, and 60% (50.0–78.8) at 6 months. No statistically significant differences were found between any of the time points (all adjusted *p* > 0.20).

One patient discontinued the study after the 2-week follow-up and did not complete subsequent scheduled assessments. This patient was monitored for safety only. According to self-reported outcomes, no systemic or local adverse events and no new complaints were recorded during this limited follow-up period.

Within the first two hours post-procedure, two patients experienced transient dysuria and another developed minimal hematuria; both events resolved spontaneously without the need for intervention. The procedure was performed without any technical difficulties in all patients, and no systemic or long-term local adverse events were reported throughout the follow-up period across all 11 patients included in the study. The results are presented in Table 1.

**Table 1:** Comparison of Preoperative and Postoperative Parameters (Friedman Test and Post-hoc Analysis)

Variable	Preop	1st Month	3rd Month	6th Month	Friedman Test (*p*)	Significant Post-hoc Result
Qmax (ml/s)	8.9 (5.3–9.8)	16.4 (11.7–23.6)	13.4 (9.1–20.2)	9.2 (6.2–14.7)	0.033	3rd month – 6th month (*p* = 0.048)
Qmean (ml/s)	5.4 (3.1–6.1)	10.2 (6.1–14.1)	7.7 (5.0–9.5)	5.8 (3.5–7.9)	0.253	None
VV (ml)	290.0 (205.2–391.0)	356.5 (329.0–400.5)	394.0 (299.5–443.8)	282.5 (191.5–476.0)	0.782	None
PMR (ml)	39.5 (22.5–79.5)	0.0 (0.0–17.5)	0.0 (0.0–35.8)	26.0 (12.5–74.8)	0.002	None
IPSS	21.0 (18.0–26.8)	13.5 (9.2–16.8)	12.5 (9.5–15.8)	15.0 (10.5–16.8)	0.006	Trend toward improvement at 3rd month (*p* = 0.035)
IIEF	4.0 (1.0–14.2)	1.0 (1.0–12.0)	3.0 (1.0–14.5)	5.0 (1.0–13.5)	0.750	None
USS-PROM Total	15.0 (13.2–19.0)	5.5 (4.2–11.0)	10.0 (7.5–10.0)	10.0 (9.2–11.5)	< 0.001	All (1st, 3rd, and 6th months < Preop)
Q8 (Voiding Picture)	3.0 (3.0–3.0)	2.0 (2.0–2.8)	2.0 (2.0–2.8)	3.0 (2.0–3.0)	0.0066	None
Patient Satisfaction (%)	45.0 (36.2–72.5)	77.5 (64.0–87.5)	70.0 (52.5–80.0)	60.0 (50.0–78.8)	0.058	None

*Qmax* Maximum urinary flow rate, *Qmean* Mean urinary flow rate, *VV* Voided volume, *PMR* Post-micturition residual urine, *IPSS* International Prostate Symptom Score, *IIEF* International Index of Erectile Function, *USS-PROM* Urethral Stricture Surgery – Patient-Reported Outcome Measure, *Q8* Voiding picture score (1 = best, 4 = worst)

## Discussion

This pilot study presents the first clinical experience with mesenchymal stem cell (MSC) therapy as an adjunctive treatment for recurrent bulbar urethral stricture. Although no statistically significant improvement was demonstrated in objective uroflowmetry parameters (Qmax and Qmean), patient-reported outcomes such as IPSS and USS-PROM showed short-term improvements, particularly within the first three months. Importantly, no local or systemic adverse events were observed in any of the patients, confirming the favorable safety profile of the procedure.

Injection techniques are widely used in urology due to their ease of application and effectiveness in treating conditions such as stress urinary incontinence, overactive bladder, and bladder pain syndrome. The simplicity of these methods allows experienced clinicians to perform them routinely, making these minimally invasive procedures a preferred choice in urological practice [25–27]. In the present study, the procedure was completed in all patients within 3 to 6 min without any technical complications, demonstrating that this routinely used urological method can also be effectively applied in the treatment of urethral stricture.

The pathophysiology of urethral stricture is characterized by progressive fibrosis of the corpus spongiosum, driven by epithelial injury, urine extravasation, and dysregulated wound healing processes. Histopathological studies have shown an altered extracellular matrix composition, characterized by an increased type I/III collagen ratio [25], reduced neuronal nitric oxide synthase activity [26], and increased connective tissue growth factor expression [27], all of which contribute to the formation of dense scars and luminal narrowing. These findings underscore the central role of fibrosis in stricture pathogenesis and provide a rationale for antifibrotic interventions [28].

Preclinical studies support this therapeutic rationale. In rat models of urethral stricture, adipose-derived MSCs were shown to reduce fibrosis, preserve urethral architecture, and enhance nitric oxide synthase expression [17]. Similarly, exosomes derived from TNF-alpha–primed MSCs demonstrated superior antifibrotic effects compared to unconditioned MSCs in mouse models. Collectively, these studies highlight the potential of MSC-based therapies to mitigate fibrosis and promote functional tissue repair in the urethra [29].

An important aspect of this study was the decision to perform MSC injection 3–5 days after urethral dilatation rather than immediately. This interval was chosen based on the biology of wound healing. Tissue repair progresses through overlapping phases of hemostasis, inflammation, proliferation, and remodeling. The acute inflammatory phase, which predominates during the first 48–72 h, is characterized by the infiltration of neutrophils and macrophages, the release of reactive oxygen species, and the production of pro-inflammatory cytokines. These conditions may be unfavorable for stem cell engraftment, survival, and paracrine activity. In contrast, the proliferative phase, which begins around day 3, involves the migration of fibroblasts, angiogenesis, and extracellular matrix remodeling, thereby creating a more receptive microenvironment for MSCs. Preclinical studies have suggested that MSCs exert their maximal antifibrotic and immunomodulatory effects in the proliferative rather than the acute inflammatory phase of healing [30, 31]. Therefore, the 3–5 day interval between dilatation and injection in our protocol was designed to exploit this biological window, potentially enhancing the therapeutic impact of MSCs.

Another advantage of this approach is that Wharton’s jelly-derived MSCs were used as the cell source. Unlike autologous protocols that require harvesting of adipose tissue, bone marrow, or bladder biopsies, this strategy avoided any additional invasive procedure. Consequently, no donor-site morbidity was encountered, and patients were spared the risks and discomfort associated with harvesting ancillary tissue. This practical advantage further supports the clinical feasibility and patient acceptability of Wharton’s jelly-derived MSC therapy in urethral stricture disease [14].

Although no prior human studies have specifically evaluated MSCs for urethral stricture, multiple clinical trials have established the safety and feasibility of MSC injections in related urological conditions. In stress urinary incontinence, autologous MSC injections into the urethral sphincter have shown functional improvements with no significant safety concerns [32–34]. A study by Garcia-Arranz et al., using a comparable endoscopic injection approach, reported subjective improvement rates of 70–80% and no significant adverse events, further supporting the translational potential of MSC therapy in lower urinary tract disorders [33].

Alternative adjunctive therapies, such as mitomycin C, platelet-rich plasma, and drug-coated balloons, have been explored with mixed results and have not achieved widespread adoption [8–11]. Compared with these approaches, MSC therapy may offer a biologically targeted strategy by addressing the underlying fibrotic mechanisms rather than only providing mechanical relief [10].

In future studies, efficacy may be enhanced by increasing the number of transplanted cells or by repeating MSC injections at defined intervals. While the optimal dose, frequency, and delivery protocol remain to be determined, such strategies could augment the antifibrotic and regenerative effects observed in preclinical models. Notably, the present study has already demonstrated that intralesional MSC administration is technically feasible and safe in humans, thus providing a solid foundation for future dose-optimization and repeated-administration trials [24].

This study has several limitations. Primarily, the sample size was small, and the follow-up was limited to six months, which restricted the ability to detect longer-term outcomes. Given the chronically recurrent nature of urethral strictures, longer follow-up outcomes will be required in future studies to fully evaluate the long-term durability and efficacy of this novel MSC treatment. Furthermore, while our initial inclusion criteria were carefully defined, the broader clinical application of this technique will undoubtedly necessitate stringent patient selection. It will be critical for future larger-scale trials to meticulously identify which patient subgroups—based on stricture etiology, length, and the number of previous interventions—benefit most from this adjunctive therapy. One patient discontinued after two weeks and, although monitored for safety, did not complete efficacy assessments. The absence of a control group prevents definitive conclusions regarding efficacy. Furthermore, the optimal MSC source, cell dose, injection volume, and number of administrations remain to be determined, emphasizing the need for standardized protocols in future trials [35].

Despite these limitations, the study demonstrates that intralesional MSC injection after urethral dilatation is safe and feasible in humans. These findings warrant further investigation in larger, controlled clinical trials with extended follow-up to determine the efficacy and durability of MSC therapy in urethral stricture disease.

## Conclusion

This first-in-human pilot study demonstrates that injection of mesenchymal stem cells for recurrent bulbar urethral stricture is safe and feasible, with no local or systemic adverse events observed throughout follow-up. While symptomatic improvements were noted in patient-reported outcomes, objective measures such as uroflowmetry showed only limited benefit. These results provide a strong safety signal, supporting further clinical development of this approach. Larger, controlled studies with longer follow-up are warranted to confirm efficacy, optimize treatment protocols, and better define the role of stem cell therapy as an adjunct in the management of urethral stricture disease.

## Data Availability

All relevant data supporting the findings of this study are available from the corresponding author upon reasonable request.
